# Physical Activity Levels and Perceived Changes in the Context of Intra-EEA Migration: A Study on Italian Immigrants in Norway

**DOI:** 10.3389/fpubh.2021.689156

**Published:** 2021-07-07

**Authors:** Giovanna Calogiuri, Alessio Rossi, Laura Terragni

**Affiliations:** ^1^Department of Nursing and Health Sciences, Faculty of Health and Social Sciences, University of South-Eastern Norway, Drammen, Norway; ^2^Department of Public Health and Sport Sciences, Faculty of Health and Social Sciences, Inland Norway University of Applied Sciences, Elverum, Norway; ^3^Department of Computer Science, University of Pisa, Pisa, Italy; ^4^Department of Nursing and Health Promotion, Faculty of Health Sciences, Oslo Metropolitan University, Oslo, Norway

**Keywords:** health behavior, Italian immigrants, immigration health, exercise, social determinants of health

## Abstract

As mobility within the European Economic Area (EEA) is on the rise, it is important to understand migrants' health-related behaviors (such as physical activity [PA]) within this context. This study investigated i) the extent to which Italian immigrants in Norway perceive that moving had a negative or positive impact on their PA; ii) possible differences between the PA of the Italian immigrants compared with the Norwegian population; and iii) possible associations of the Italian immigrants' PA with key sociodemographic characteristics (gender, age, region of residence, and educational level). The data were retrieved from the *Mens Sana in Corpore Sano* study. In order to enhance the sample's representativeness, the original dataset (*n* = 321) was oversampled in accordance with the proportion of key sociodemographic characteristics of the reference population using the ADASYN method (resampled *n* = 531). The results indicate that a large majority of Italian immigrants perceived that they were as or even more physically active in Norway than they would have been if they continued living in Italy, while 20% of the Italians perceived instead a negative impact. No significant differences were found in the PA levels of the Italians in comparison with the Norwegian population, though some differences were found in relation to specific modes of PA. After controlling for multiple sociodemographic characteristics, men, those with lower educational levels and, to a certain extent, older adults tended to perceive a more negative impact and be less physically active than their respective counterparts. Compared with those living in the most urbanized regions, a larger proportion of those living in less urbanized regions perceived a negative impact, though no differences were observed in terms of PA levels. The findings are discussed in light of acculturation, gender, and social gradient. The knowledge generated by this study sheds light on an important health-related behavior among Italians in Norway, which can inform initiatives that aim at promoting PA in this specific group as well as other similar contexts of intra-EEA migration.

## Introduction

### The Salutogenic Effects of Physical Activity

Physical activity (PA) is one of the lifestyle factors that most influence whether people live a long and healthy life. Recent meta-analyses based on studies using self-reported (1) or accelerometry-based assessments (2) have provided yet again clear evidence that greater amounts of physical activity (regardless of intensity) and lesser time spent in sedentary behavior are associated with a lower risk for premature mortality. Not only an active lifestyle makes people live longer, it also contributes to better mental health (3, 4) and, in general, a higher quality of life (5). Furthermore, recent analyses have emphasized the significance of PA as a sustainable behavior that can relate to several of the United Nations' sustainable development goals (6).

Health-enhancing PA includes any bodily movement that leads to an increase in energy expenditure (7). This definition includes, but it is not limited to, structured physical exercise (for example, when one exercises in the gym or plays a sport). However, activities such as walking, doing house chores, occupational activities that require physical movement, and many other non-structured activities can also be construed health-enhancing PA. Indeed, according to the latest recommendations by the World health Organization (WHO), every minute spent in PA counts (7). More specifically, the WHO recommends that, in order to improve and/or maintain good psycho-physical health, adults and elderly should engage in aerobic PA of light- or moderate-intensity (e.g., walking, cycling, swimming) for at least 150 min per week, or in aerobic PA of vigorous-intensity (e.g., running) for at least 75 min per week, or an equivalent combination of light-/moderate- and vigorous-intensity PA. The WHO also recommends performing exercises aimed at increasing muscular strength, flexibility and balance. Moreover, it is recommended to avoid, for as much as possible, to spend prolonged time in inactivity or sedentary behaviors (e.g., sitting to work or watching TV).

In spite of the consistent evidence on the health benefits of PA, as well as the clear and simple guidelines for PA behavior, insufficient PA remains one of the leading risk factors for poor health and mortality worldwide (7). In a health promotion perspective, a major challenge in promoting PA is that this behavior is subjected to social gradients, with more vulnerable sub-groups of the population less likely to engage in sufficient PA levels or to respond to PA promotion initiatives. In particular, studies have consistently shown that gender, age, educational level, and ethnic background are major social determinants of PA behavior (8–10).

### Physical Activity in Norway

Compared to other western countries, Norwegians are estimated to be relatively active. Figures from the Global Health Observatory (11) indicate that, in 2016, 68% of adult Norwegians met the WHO's recommended levels for PA. This prevalence was higher compared with other European countries such as, for example, Italy, where 59% of the adults engaged in sufficient PA levels. Moreover, compared with other countries, Norway shows a smaller gender-gap: while in Italy 54% of the men and 44% of women met the PA recommendations, in Norway 70% of men and 66% of women met the WHO's PA recommendations (11). National studies based on both, self-reported and objective assessments of PA, indicate that the gender-gap in Norway may be even smaller (12, 13). The abundance of and generally good accessibility to safe natural environments, as well as the general cultural attitude that values PA and outdoor recreations, have been suggested as important factors favoring higher PA levels in the Norwegian population (14, 15). In spite of this, the Norwegian population is not exempt from PA-related social gradients. In particular, national studies based on both subjective and objective assessments showed that the prevalence of people who engage in sufficient amounts of PA increase with increasing educational level (13). The region of residence, and especially whether one lives in a more or less urbanized setting, is also known to influence Norwegians' PA patterns, especially in relation to specific PA modes. For example, exercising in the gym and active transport were found to be more common among people who live in more urbanized areas (14). Although the gender-gap for PA is relatively small, some differences between men and women still exist: for instance, in 2019, slightly fewer women than men (53 and 56%, respectively) were reported to engage in moderate-to-vigorous PA (MVPA) for at least 150 min during regular week (12).

Evidence exists also on PA differences between the Norwegian general population and individuals with immigration background. Immigrants are defined as persons born abroad by foreign-born parents (16). According to a 2016 survey on the living conditions of different immigrant groups (prevalently from a non-western countries), 57% of the immigrants engaged in PA once or more times during a regular week, as opposed to 71% for the Norwegian general population in the same year (17). The survey also found a larger gender-gap among the immigrants compared with the Norwegian general population: the proportion of men and women immigrants engaging in PA once or more times a week was 61 and 53%, respectively, whereas it was 69 and 73%, respectively, in the Norwegian general population (17). However, it should be noted that, to the best of the authors' knowledge, all existing studies on PA among immigrants in Norway focused on groups from non-western countries, while the composition of the immigrant population living in Norway is more heterogeneous, with most immigrants coming from Europe (18).

### Italian Immigrants in Norway

Italian immigrants in Norway, although still relatively low in number, are a rapidly growing group. Italians enjoy the right of *Free movement of people* within the European Union and the European Economic Area (EEA; which includes Norway). Italians' immigration to Norway has been steadily increasing since the establishment of the EEA Agreement in 1994, and it has 3-fold since the economic crisis of 2008 (19, 20). This trend is in line with the increased Italian mobility worldwide: from 2006 to 2019, the number of Italians registered as residents abroad increased by 70%, going from over 3.1 million to almost 5.3 million (19). These new waves of Italian immigrants have been often described as “the better youth” (young, well-educated, cosmopolitan and mobile individuals), thought there are also indications that grownups and families have been moving looking for job or better living conditions (20). According to figures from the Italian Embassy in Norway, to date 7.108 Italian citizens reside in Norway and are registered at the Norwegian Register of Italians Living Abroad (AIRE). Of these, 4.523 (2.862 men and 1.661 women) are Italian-born, while the other are progenies of Italian immigrants (20). In spite of this rapid increment, the living conditions of this group, especially in relation to their health and health-related behaviors such as PA, have received virtually no attention.

### Purpose of the Study

Knowledge about the PA habits of the Italian immigrants can provide novel insight on the health and lifestyle of this under-researched immigration group. Moreover, investigating the PA habits of this group, which has substantially different characteristics from previously studied groups (especially, immigrants from non-western countries), can shed new light into factors influencing health behaviors among immigrant population in general. Thus, the purpose of this study was to investigate the extent to which Italian immigrants in Norway perceive that moving to Norway had a negative or positive impact on their PA habits, as well as investigate whether (and how) the PA profile of the Italians differ from the general Norwegian population. Furthermore, the study aimed to investigate whether (and how) the PA profile of the Italians varied in relation to key sociodemographic characteristics.

The following research questions (RQ) were designed to guide the study:

RQ1. To what extent do Italian immigrants in Norway perceive that moving to Norway had a negative or positive impact on their PA habits?
RQ2. Does the PA profile (in terms of daily time spent sitting, weekly MVPA amount, weekly MVPA frequency, and preferred modes of MVPA) of Italian immigrants in Norway differ compared with the general Norwegian population?
RQ3. How does the PA profile of Italian immigrants in Norway vary in relation to gender, age, region of residence, and educational level?

## Methods

### Data Collection and Participants

The data for this study were retrieved from the Mens Sana in Corpore Sano study (21), an investigation on health-related behaviors among Italian immigrants in Norway. The study was conducted in collaboration with Comites Oslo (an elected representative body of the Italian community) and the Italian Embassy in Norway. An online questionnaire was distributed during the period between 15 March and 24 April 2019 (hence, before the lockdown due to the COVID-19 pandemic). As it was not possible to access a reliable list with the contact information of all the Italians living in Norway, the survey was distributed through different channels, including mail-lists of associations of Italians in Norway, adverts on the websites of Comites Oslo's and the Italian Embassy's, as well as different Facebook groups for Italians in Norway. Respondents were also invited to forward the invitation to friends and acquaintances according to a snowball sampling strategy. All Italian-speaking immigrants, aged 18 years or older, resident in Norway at the time of the survey and who spent most of their childhood (up to age 16 years) in Italy were invited to participate in the survey. To assure that the respondents met these criteria, a set of control questions was introduced at the beginning of the survey. A total of 330 people responded to the survey, of whom 321 met all inclusion criteria (see Table 1 for descriptive statistics of the respondents).

**Table 1:** Data distribution of the reference population, the original dataset, and the resampled dataset.

**Sociodemographic characteristics**	**Italians in Norway** **(*N* = 3,474)^**a**^**	**Mens Sana in** **Corpore Sano dataset** **(*n* = 321)**	**Resampled dataset** **(*n* = 531)^**b**^**
**Gender**			
Male	63%	47%	61%
Female	37%	53%	39%
**Age**			
18–30 y	23%	14%	19%
31–50 y	54%	71%	56%
≥50 y	23%	15%	25%
**Educational level (highest completed)**			
Up to secondary upper-level school	40%	18%	39%
Bachelor or higher	60%	82%	61%
**Region of residence**			
North	3%	7%	6%
Center	7%	11%	12%
West	20%	14%	19%
Oslo/Akershus	53%	60%	57%
Other eastern regions	13%	8%	6%
South	3%	1%	1%

a *Based on the AIRE register; information provided by the Italian Embassy in Norway (22)*.

b *Resampling was based on Age and Educational level*.

*Region of residence: North = Finnmark, Troms, Nordland; Center = N. & S.Trøndelag, Møre og Romsdal; West = Sogn og Fjordane, Hordaland, Rogaland; Other eastern regions = Telemark, Buskerud, Vestfold, Østfold, Oppland, Hedmark; Sout = V. Agder, A. Agder*.

### Oversampling

A comparison of basic sociodemographic characteristics (gender, age, educational level, and region of residence) of the sample with figures provided by national registers, such as AIRE and Statistics Norway (16), revealed that our sample was not fully representative of the overall population of Italian immigrants in Norway. The sample had a larger proportion of women, mid-aged individuals, people with a higher educational level, and people living in the region of Oslo-Akershus, which is the most densely populated and urbanized area of Norway (Table 1). In order to enhance the sample's representativeness, the dataset was oversampled in accordance with the proportion of key sociodemographic characteristics of the reference population, which were provided by the Italian Embassy as based on the AIRE registry. To this aim, Adaptive Synthetic Sampling Method for Imbalanced Data (ADASYN) was applied (23) by using the publicly available Python package imblearn (http://scikit-learn.org/imbalanced-learn). ADASYN automatically estimates the needed number of synthetic cases according to a density distribution defined by the expected proportions of given variable in the reference population of Italians in Norway. In particular, considering a vector case *x*_*i*_, a new vector case *x*_*new*_ will be generated considering its *k* 3 nearest-neighbors (*x*_*zi*_) as follow:

$$\begin{array}{l} X_{new}=x_{i}+\lambda \;*\;\left(x_{zi}-x_{i}\right) \end{array}$$

where λ is a random number between [0,1]. For this study, the oversampling process was performed on 2-factors, i.e., age and educational level. The resampled dataset reflects an acceptable distribution of key sociodemographic variables with relatively limited inclusion of synthetic cases (additional synthetic cases = 231; overall *n* = 531; Table 1).

### Instruments

The questionnaire used in the Mens Sana in Corpore Sano study (which was in Italian language) was developed in order to allow comparisons with existing surveys in the Norwegian population. More specifically, the items were taken from or closely inspired to items used in the following surveys:

- *Levekårundesøkelsen om helse* (Survey on living conditions), a routinely conducted survey on health, health-related behaviors, and social relations in the general Norwegian population [description and aggregated data available in Statistics Norway, 2019 (12)];
- *FRIFOs aktivitetskartlegging 2012* (Physical activity survey for FRIFO 2012), a survey on physical activity behaviors and motivations in the general Norwegian population initiated by The Norwegian Outdoor Council in 2012 [description and aggregated data available in Calogiuri et al. (14)].

For the present study, the following variables were used:

*Perceived impact of moving -* This variable provided an indication of the extent to which, all in all, the respondents perceived that their PA habits were influenced (positively or negatively) by the migration process. This was measured with a single item inquiring the following: “Imagine that you did not move to Norway and, instead, continued to live in Italy. What of the following statements would better reflect your physical activity habits in such an hypothetical circumstance?” The response options were: “I would have been more physically active in Italy than I am now in Norway,” “I would have been as physically active in Italy as I am now in Norway,” and “I would have been less physically active in Italy than I am now in Norway.” The respondents who selected the first response option were considered as perceiving a negative impact on their PA habits, those who selected the second response option were considered as perceiving no significant impact, while those who selected the third response option were considered as perceiving a positive impact. An additional response option “I don't know” was also included, but was excluded from further analyses.

*Time spent sitting -* This was assessed through a single item inquiring “During a regular day, on average, how many hours do you spend sitting?,” with the response options being “Less than 6 h,” “Between 6 and 9 h,” “More than 10 h.”

*Weekly amounts of moderate-to-vigorous physical activity (MVPA amount) -* This variable provided an indication of the extent to which the respondents engaged in insufficient or sufficient PA levels, broadly in line with the WHO's recommendations. First, the following definition of PA was presented: “Think about your physical activity habits in the course of the past 12 months. By the term “physical activity” we intend any bodily movement that, for at least 10-consecutive minutes, makes your heart beat faster and your breathing harder. This can include, for example, structured exercise but also activities during working- or school hours, house chores, going for a stroll, or if you walk or bike to/from work.” Subsequently, the following question was presented “During a regular week, all in all, how much time do you spend doing physical activity?” with the response options being “I never engage in any MVPA during a regular week,” “Less than 2.5 h,” “Between 2.5 and 5 h,” and “More than 5 h.”

*Weekly frequency of moderate-to-vigorous physical activity (MVPA frequency) -* The following question was presented: “During a regular week, how often do you engage in physical activity as described above?” (referring to the definition provided in the previous section). The respondents were then asked to select one of the following response options: “Never/less than once a week” or “Once or more times a week.” If they selected the latter option, they were asked to specify the number of times by selecting from a list a number between 1 to 6, or an additional option “7 or more.”

*Preferred modes of moderate-to-vigorous physical activity (MVPA modes) -* This variable was used to provide an indication of the activities the respondents engaged in during a regular week, both in binary terms (i.e., whether or not they participated in a given activity) as well as in relative terms (i.e., time spent in a given activity as relative to the overall weekly PA). The following question was posed: “Think about the overall time that, normally, you spend doing physical activity. More or less, how much of this time do you spend in each of the following activities?” Then the respondents were presented with a list of activities, each accompanied by a brief explanation:

- “Organized sports (for example, football, volleyball, track-and-field, etc.).”
- “Exercising in the gym (for example, fitness group-classes, weightlifting, etc.).
- “Occupational activity (for example, working activities that require some physical exertion, sports or activity within school hours, etc.).”
- “Active transport (that is walking, biking, skate-boarding, etc. to/from destinations such as your workplace).”
- “Green exercise (walking or other exercise in parks, green areas, or other natural environments).”
- “Other activities.”

For each of these activities, the respondents had to select one of the following options: “Not even a bit,” “Less than half of the overall time,” “About half of the overall time,” “More than half of the overall time,” or “I only do this activity.” As the purpose of the study was to investigate which activities most contributed to the Italians *weekly* PA, this item was visible only to the respondents who in the MVPA frequency item selected the option “Once or more time a week,” while those who reported that they engage in PA never or less than once a week were automatically coded as spending “Not even a bit” of their weekly PA for all of the activities.

*Sociodemographic characteristics –* Key sociodemographic variables were collected in order to investigate possible social gradients in relation to the Italian immigrants' PA profile. This included:

- *Gender* (male; female),
- *Age* (<30 yr; 30–39 yr; 40–49 yr; ≥50 yr)
- *Region of residence* (as an indicator of the respondents living environment, especially with respect to centrality/urbanization). For the purposes of this study, Region of residence, which in the Mens Sana in Corpore Sano survey was assessed by grouping Norway's majors geographical areas (see Table 1), was re-coded as the respondents living in “Oslo/Akershus” (which is the most urbanized and densely populated region of Norway) or any other region of Norway. Although this resulted in a rather imprecise indicator of the Italian's living environment, this was done as most respondents do live in the region Oslo/Akershus (see Table 1), while all other regions were rather little represented in comparison.
- *Educational level* (as an indicator of socio-economic status [SES]), with reference to the highest attained (“High-school or lower,” “Bachelor's or Master's Degree,” or “Doctoral Degree”).

### Statistical Analyses

All the analyses in this study were conducted by an experienced data scientist using Python 3.8 programming language. An exploratory analysis was preliminary conducted in order to examine the data distribution and tentatively investigate possible associations among the variables. Possible interactions among different variables selected were also explored through examination of a correlation matrix and correlation network (Supplementary Figures 1, 2, and Supplementary Table 1). This process led to identification of the variables utilized in the study. In order to address RQ1 and RQ2, descriptive analyses (percentages) were performed on all PA variables (Perceived impact of moving, Daily time spent sitting, MVPA amount, MVPA frequency, and all the MVPA modes), as based on the resampled dataset. One sample Chi-squared (χ^2^) tests were performed to compare the observed proportion of the PA variables with the figures available for the Norwegian population, as provided in form of aggregated data by Statistics Norway (12) and in Calogiuri et al. (14). The variables of the study were re-coded and adapted in such way to allow direct comparisons with available figures for the Norwegian population. To address RQ3, firstly, a set of one-way Analysis of Variance (ANOVA) tests were performed to investigate whether, and to what extent, the PA variables were individually associated with the sociodemographic variables (unifactorial model). The different PA variables (Perceived impact, Time spent sitting, MVPA amount, MVPA Frequency, and the different MVPA modes) were individually set as dependent variable, while the sociodemographic variables (gender, age groups, region of residence, and educational level) were set as individual factors. Subsequently, for each of the PA variables, a four-ways ANOVA was performed including all sociodemographic variables in a full factorial model (multifactorial model), in order to establish whether possible significant associations were maintained when controlling for multiple sociodemographic variables. As the output (dependent variables) were all at a categorical-ordinal level, all ANOVA analyses were programmed based on Ordinal Regression. In particular, using Python 3.8 language programming, the Ordinal Regression was fitted for each output (the model for Ordinal Regression was specified using formula notation), and F statistics (ANOVA table) were obtained from the regression analysis result. If statistical significance was achieved in the multifactorial ANOVA, a χ^2^ test for independence was performed as a *post-hoc* analysis. By testing all combinations of between-groups comparisons, this approach allowed emphasizing possible differences among sub-groups of the sample. The statistical significance was set at *p* <0.05.

## Results

### Perceived Impact of Moving

Twenty percent of the Italians perceived that they were less physically active in Norway than they would have been if they continued living in Italy (negative impact), while 35% perceived they were as physically active in Norway as they would have been if they continued living in Italy (no relevant impact), and 37% perceived that they were more physically active in Norway than they would have been if they continued living in Italy (positive impact). Eight percentage reported they could not tell.

### Comparison With the Norwegian Population

**Figure 1:**
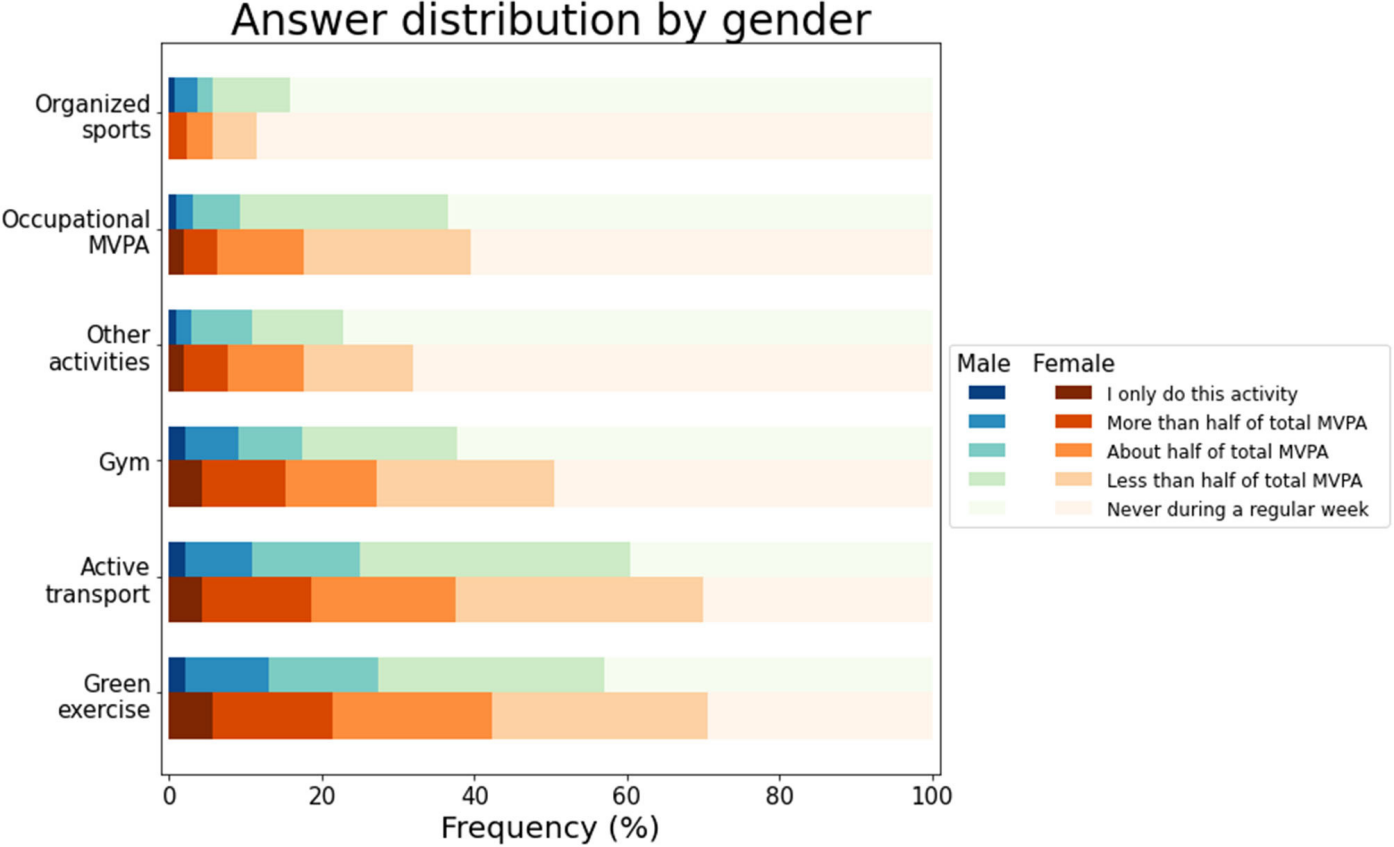
Prevalence of male and female Italian immigrants in Norway engaging in different modes of MVPA during a regular week relative to their total MVPA.

Descriptive and **χ^2^** statistics comparing the PA profile of the Italian immigrants with the Norwegian general population are presented in Table 2. The large majority of the Italians (93%) spent 9 h/day or less sitting, while 7% spent 10 h/day or more sitting. The comparison with the figures provided by the 2019 Survey on living conditions in the general Norwegian population [Statistics Norway, 2020 (12)] showed that the prevalence of Italians and Norwegians spending 10 h/day or more sitting (8%) were rather similar, with no statistically significant difference being detected. Fifty-four percent of the Italians engaged in at least 150 MVPA min/week, a prevalence that was similar to the general Norwegian population (55%). In average, the Italians engaged in MVPA 2.68 ± 2.22 times during a regular week, with 77% of the Italians engaging in some MVPA at least once during a regular week. This prevalence was similar compared with the general Norwegian population's (74%). The most popular modes of MVPA reported by the Italians were Green exercise, Active transport, and Exercising in the gym. Not only were these activities the most popular, but they also appear to contribute substantially to the Italians' weekly PA routines, as indicated by the fact that for a relatively large portion of the sample (30, 33, and 21%, respectively) these activities covered half-part or more of the total weekly MVPA (Figure 1). Occupational MVPA and Other activities were also relatively popular, while Organized sports was the least practiced activity. The comparison of these findings with figures from the Physical activity survey for FRIFO 2012, as retrieved from Calogiuri et al. (14), showed that the proportion of Italians engaging in Green exercise during a regular week was similar compared with the Norwegian population (64 vs. 62%). Similar patterns were found also for Organized sports (14 vs. 15% for Italians and Norwegians). On the other hand, significantly larger proportions were found among the Italians, as compared with Norwegians, for Active transport (62 vs. 26%), Exercising in the gym (43 vs. 24%), and Other activities (38 vs. 8%). A marginally significant difference was found for Occupational MVPA (26 vs. 19%).

**Table 2:** Physical activity profile of Italian immigrants in Norway compared with the Norwegian general population.

**PA variables**	**Italian** **immigrants^**a**^**	**Norwegian** **population^**b**^**	**One-sample χ^2^ test**
**Time spent sitting**			*χ^2^ =* 0.09; *p =* 0.77
<10 h/day	93%	92%	
≥ 10 h/day	7%	8%	
**MVPA amount**			*χ^2^ =* 0.08; *p =* 0.78
<150 min/week	46%	45%	
≥150 min/week	54%	55%	
**MVPA frequency**			*χ^2^ =* 0.35; *p =* 0.56
Never during a regular week	23%	26%	
Once or more times a week	77%	74%	
**Green exercise**			*χ^2^ =* 0.18; *p =* 0.67
Never during a regular week	36%	38%	
On a weekly base	64%	62%	
**Active transport**			*χ^2^ =* 71.66; *p* <0.001
Never during a regular week	38%	76%	
On a weekly base	62%	24%	
**Exercise in the gym**			*χ^2^ =* 14.50; *p* <0.001
Never during a regular week	57%	74%	
On a weekly base	43%	26%	
**Occupational MVPA**			*χ^2^ =* 3.56; *p =* 0.05
Never during a regular week	62%	81%	
On a weekly base	38%	19%	
**Organized sports**			*χ^2^ =* 0.06; *p =* 0.80
Never during a regular week	86%	85%	
On a weekly base	14%	15%	
**Other**			*χ^2^ =* 119.85; *p* <0.001
Never during a regular week	74%	92%	
On a weekly base	26%	8%	

a *Based on oversampled dataset (n = 531)*.

b *Figures retrieved from Statistics Norway, 2020 (12), and Calogiuri et al. (14)*.

### Physical Activity Profile Across Sociodemographic Characteristics

Statistics for all the PA values in relation to Gender are presented in Table 3. Statistically significant differences between men and women were found for the perceived impact of moving, with a larger proportion of women perceiving that they were more active in Norway than they would have been if they continued living in Italy, while a larger proportion of men reported a negative impact. The association was reduced, but it remained statistically significant, in the multivariate model controlled for age, region of residence, and educational level. No statistically significant differences between men and women were found for Time spent sitting. On the other hand, women emerged as being generally more physically active than men, showing significantly higher levels of MVPA amount and MVPA frequency (both significant before and after controlling for multiple sociodemographic variables). Moreover, a significantly larger proportion of women, as compared with men, reported to engage in Green exercise, Active Transport, and Exercise in the gym during a regular week (all significant before and after controlling for multiple sociodemographic variables). No significant differences between men and women were found for Organized sports, Occupational MVPA, or Other activities.

**Table 3:** Physical activity profile of Italian immigrants in Norway, by gender (*n* = 531).

**Physical activity variables**	**Gender**		**1-way ANOVA** **(unifactorial model)**	**4-way ANOVA** **(multifactorial model)**
	**Men** **(*n* = 321)**	**Women** **(*n* = 210)**		
**Perceived impact**	b	a	F_(1, 529)_ = 8.64 ^***^	F_(1, 515)_ = 4.56 ^*^
Negative	24.4%	18.6%		
No impact	42.9%	29.9%		
Positive	32.7%	51.5%		
**Time spent sitting**	n.s.	n.s.	F_(1, 529)_ = 0.75	F_(1, 515)_ = 0.71
<6 h/day	41.7%	37.7%		
6–9 h/day	52.1%	53.8%		
≥ 10 h/day	6.2%	8.5%		
**MVPA amount**	b	a	F_(1, 529)_ = 4.22 ^**^	F_(1, 515)_ = 7.77 ^**^
Never during a regular week	27.4%	16.7%		
<150 min/week	24.6%	21.0%		
150–300 min/week	29.6%	39.1%		
> 300 min/week	18.4%	23.2%		
**MVPA frequency**	b	a	F_(1, 529)_ = 2.31 ^*^	F_(1, 515)_ = 8.80 ^***^
M ± SD	2.74 ± 2.20	3.34 ± 2.21		
**Green exercise**	b	a	F_(1, 529)_ = 2.65 ^*^	F_(1, 515)_ = 4.75 ^*^
Not on a weekly base	39.6%	30.0%		
On a weekly base^c^	60.4%	70.0%		
**Active transport**	b	a	F_(1, 529)_ = 4.02 ^**^	F_(1, 515)_ = 9.16 ^***^
Not on a weekly base	43.0%	29.5%		
On a weekly base^c^	57.0%	70.5%		
**Exercise in the gym**	b	a	F_(1, 529)_ = 2.42 ^*^	F_(1, 515)_ = 2.21 ^*^
Not on a weekly base	62.3%	49.5%		
On a weekly base^c^	37.7%	50.5%		
**Occupational MVPA**	n.s.	n.s.	F_(1, 529)_ = 2.11	F_(1, 515)_ = 2.17
Not on a weekly base	77.3%	68.1%		
On a weekly base^c^	22.7%	31.9%		
**Organized sports**	n.s.	n.s.	F_(1, 529)_ = 1.88	F_(1, 515)_ = 1.91
Not on a weekly base	84.1%	88.6%		
On a weekly base^c^	15.9%	11.4%		
**Other**	n.s.	n.s.	F_(1, 529)_ = 1.91	F_(1, 515)_ = 2.13
Not on a weekly base	63.6%	60.5%		
On a weekly base^c^	36.4%	39.5%		

*1-way ANOVA = Gender included as single factor; 4-way ANOVA = Gender included as factor while correcting for other sociodemographic variables (Age, Educational level, and Region of residence) in a full factorial 4-ways ANOVA*.

*** *p <0.001;*

** *p <0.01;*

* *p <0.05*.

a−b *χ^2^ test for independence showed a significant difference (p <0.05): (a) vs. group 1; (b) vs. group 2*.

c *Original levels “Less than half of total MVPA,” “About half of total MVPA,” “More than half of total MVPA,” and “I only do this activity” are presented conflated*.

Statistics for all the PA values in relation to Age are presented in Table 4. The perceived impact of moving varied significantly across age groups, with the association remaining significant when controlling for multiple sociodemographic variables. Compared with the other age groups, those aged 30–39 yr had the largest prevalence of perceiving a positive impact and lowest prevalence of perceiving a negative impact. On the other hand, compared with the other age groups, those aged ≥50 yr showed the smallest prevalence of perceiving a positive impact and the largest prevalence of perceiving a negative impact. No statistically significant differences across age groups were found for Time spent sitting or MVPA amount. Significant differences across age groups were found however for MVPA frequency (both in the unifactorial and the multifactorial model), with those aged 30–39 yr reporting the highest frequency and the Italians aged <30 yr reporting the lowest frequency, as compared with the other age groups. For what concerts the modes of MVPA, significant differences across age groups were found for Active transport (most commonly practiced by those aged 30–39 yr), Exercising in the gym (most commonly practiced by those aged 30–39 yr), and Other activities (most commonly practiced by the Italians aged ≥50 yr). The unifactorial model found a significant effect of Age also for Occupational MVPA (most commonly practiced by those aged <30 yr), though the association was no longer significant when controlling for multiple sociodemographic variables, No significant differences across age groups were found for Green exercise or Organized sports.

**Table 4:** Physical activity profile of Italian immigrants in Norway, by age (*n* = 531).

**Physical activity variables**	**Age**				**1-way ANOVA** **(unifactorial model)**	**4-way ANOVA** **(multifactorial model)**
	** <30 yr** **(*n* = 101)**	**30–39 yr** **(*n* = 165)**	**40–49 yr** **(*n* = 133)**	**≥50 yr** **(*n* = 132)**		
**Perceived impact**	b, d	a, c, d	b, d	a, b, c	F_(3, 529)_ = 6.94 ^***^	F_(3, 515)_ = 8.72 ^***^
Negative	18.8%	19.1%	26.8%	23.7%		
No impact	39.6%	29.9%	34.1%	50.9%		
Positive	41.6%	51.0%	39.1%	25.4%		
**Time spent sitting**	n.s.	n.s.	n.s.	n.s.	F_(3, 529)_ = 0.69	F_(3, 515)_ = 0.04
<6 h/day	39.6%	37.6%	38.3%	45.5%		
6–9 h/day	56.4%	52.7%	51.3%	51.5%		
≥ 10 h/day	4.0%	9.7%	10.4%	3.03%		
**MVPA amount**	n.s.	n.s.	n.s.	n.s.	F_(3, 529)_ = 0.34	F_(3, 515)_ = 0.51
Never in a regular week	25.7%	20.0%	33.3%	25.0%		
<150 min/week	24.8%	19.4%	24.8%	25.0%		
150–300 min/week	30.7%	35.8%	32.3%	33.3%		
> 300 min/week	18.8%	24.8%	19.6%	16.7%		
**MVPA frequency**	b, c, d	a, d	a, d	a, b, c	F_(3, 529)_ = 4.57 ^***^	F_(3, 515)_ = 6.23 ^***^
M ± SD	2.72 ± 1.93	3.19 ± 2.27	2.97 ± 2.27	2.91 ± 2.32		
**Green exercise**	n.s.	n.s.	n.s.	n.s.	F_(3, 529)_ = 1.79	F_(3, 515)_ = 0.18
Not on a weekly base	40.6%	32.7%	36.1%	35.6%		
On a weekly base^e^	59.4%	67.3%	63.9%	64.4%		
**Active transport**	n.s.	d	d	b.c	F_(1, 529)_ = 2.42 ^**^	F_(3, 515)_ = 4.21 ^**^
Not on a weekly base	44.6%	30.9%	36.1%	42.4%		
On a weekly base^e^	55.4%	69.1%	63.9%	57.6%		
**Exercise in the gym**	d	d	n.s.	a.b	F_(3, 529)_ = 7.41 ^***^	F_(3, 515)_ = 3.83 ^*^
Not on a weekly base	58.4%	47.9%	58.6%	66.7%		
On a weekly base^e^	41.6%	52.1%	41.4%	33.3%		
**Occupational MVPA**	c	n.s.	a	n.s.	F_(3, 529)_ = 2.96 ^**^	F_(3, 515)_ = 0.82
Not on a weekly base	68.3%	72.1%	78.9%	74.2%		
On a weekly base^e^	31.7%	27.9%	21.1%	25.8%		
**Organized sports**	n.s.	n.s.	n.s.	n.s.	F_(3, 529)_ = 0.47	F_(3, 515)_ = 2.17
Not on a weekly base	80.2%	88.5%	83.5%	89.4%		
On a weekly base^e^	19.8%	11.5%	16.5%	10.6%		
**Other**	d	n.s.	n.s.	a	F_(3, 529)_ = 3.06 ^**^	F_(3, 515)_ = 17.75 ^***^
Not on a weekly base	74.3%	62.4%	59.4%	56.1%		
On a weekly base^e^	25.7%	37.6%	40.6%	43.9%		

*1-way ANOVA = Age included as single factor; 4-way ANOVA = Age included as factor while correcting for other sociodemographic variables (Gender, Educational level, and Region of residence) in a full factorial 4-ways ANOVA.*

*** *p <0.001;*

** *p <0.01;*

* *p <0.05.*

a−d *χ^2^ test for independence showed a significant difference (p <0.05): (a) vs. group 1; (b) vs. group 2; (c) vs. group 3; (d) vs. group 4*.

e *Original levels “Less than half of total MVPA,” “About half of total MVPA,” “More than half of total MVPA,” and “I only do this activity” are presented conflated*.

Statistics for all the PA values in relation to Region of residence are presented in Table 5. Statistically significant differences were found for the Perceived impact of moving, with a larger proportion of Italians living in Oslo/Akershus perceiving a positive impact, while a larger portion of Italians living in other regions of Norway perceived a negative impact. No significant differences between regions of residence emerged for any of the other PA variables, with the exception of Occupational MVPA in the unfactorial model, which was more prevalent among those who live in other regions of Norway. This association was, however, no longer significant in the multifactorial model controlling multiple sociodemographic variables.

**Table 5:** Physical activity profile of Italian immigrants in Norway, by region of residence (*n* = 531).

**Physical activity variables**	**Region of residenc**		**1-way ANOVA** **(unifactorial model)**	**4-way ANOVA** **(multifactorial model)**
	**Oslo/Akershus** **(*n* = 300)**	**Other regions** **(*n* = 231)**		
**Perceived impact**	a	b	F_(1, 529)_ = 6.95 ^***^	F_(1, 515)_ = 11.23 ^***^
Negative	16.2%	29.4%		
No impact	40.1%	34.9%		
Positive	43.7%	35.7%		
**Time spent sitting**	n.s.	n.s.	F_(1, 529)_ = 0.36	F_(1, 515)_ = 0.02
<6 h/day	38.7%	42.0%		
6-9 h/day	54.3%	50.7%		
≥ 10 h/day	7.0%	7.3%		
**MVPA amount**	n.s.	n.s.	F_(1, 529)_ = 0.24	F_(1, 515)_ = 0.23
Never during a regular week	23.3%	22.9%		
<150 min/week	22.3%	24.2%		
150-300 min/week	34.7%	31.6%		
> 300 min/week	19.7%	21.3%		
**MVPA frequency**	n.s.	n.s.	F_(1, 529)_ = 1.38	F_(1, 515)_ = 1.51
M ± SD	2.95 ± 2.23	3.01 ± 2.22		
**Green exercise**	n.s.	n.s.	F_(1, 529)_ = 2.05	F_(1, 515)_ = 0.80
Not on a weekly base	32.0%	40.7%		
On a weekly base^c^	68.0%	59.3%		
**Active transport**	n.s.	n.s.	F_(1, 529)_ = 0.87	F_(1, 515)_ = 3.71
Not on a weekly base	35.3%	40.7%		
On a weekly base^c^	64.7%	59.3%		
**Exercise in the gym**	n.s.	n.s.	F_(1, 529)_ = 0.40	F_(1, 515)_ = 0.07
Not on a weekly base	56.0%	58.9%		
On a weekly base^c^	44.0%	41.1%		
**Occupational MVPA**	b	a	F_(1, 529)_ = 3.04 ^**^	F_(1, 515)_ = 3.40
Not on a weekly base	78.7%	67.1%		
On a weekly base^c^	21.3%	32.9%		
**Organized sports**	n.s.	n.s.	F_(1, 529)_ = 1.12	F_(1, 515)_ = 0.57
Not on a weekly base	87.7%	83.5%		
On a weekly base^c^	12.3%	16.5%		
**Other**	n.s.	n.s.	F_(1, 529)_ = 2.05	F_(1, 515)_ = 2.42
Not on a weekly base	63.7%	60.6%		
On a weekly base^c^	36.3%	39.4%		

*1-way ANOVA = Region of residence included as single factor; 4-way ANOVA = Region of residence included as factor while correcting for other sociodemographic variables (Gender, Age, Educational level) in a full factorial 4-ways ANOVA*.

*** *p <0.001;*

** *p <0.01;*

* *p <0.05.*

a−b *χ^2^ test for independence showed a significant difference (p <0.05): a) vs. group 1; b) vs. group 2.*

c *Original levels “Less than half of total MVPA,” “About half of total MVPA,” “More than half of total MVPA,” and “I only do this activity” are presented conflated*.

The findings presented in Table 6 show a rather pronounced social gradient in relation to Educational levels for all the PA variables, with the exclusion of the MVPA mode ‘Other activities'. Remarkably, the association of the PA variables with Educational levels showed, in most cases, dose-response patterns. Moreover, all associations remained significant after controlling for multiple sociodemographic variables in the multifactorial model. The prevalence of perceiving a positive impact increased with increasing educational level, while the prevalence of perceiving a negative impact was largest among the Italians with the lowest educational level. Time spent sitting increased with increasing educational levels, with those with the highest educational level showing the largest prevalence of sitting ≥10 h/day, while the prevalence of those sitting for <6 h/day increased with decreasing educational level. The prevalence of individuals engaging in higher MVPA amounts (i.e., 150–300 min/week or >300 min/week) increased with increasing educational level, while the prevalence of individuals engaging in MVPA <150 min/week or never increased with decreasing educational level. Similarly, the mean MVPA frequency, as well as the prevalence of weekly Green exercise, Active transport, and Exercising in the gym increased with increasing educational level. Quite the opposite, the prevalence of individuals engaging in Occupational MVPA decreased with increasing educational level. A marginally significant association was found between educational level and Organized sports, with the prevalence of this activity being lower in the lowest educational level as compared with the other groups.

**Table 6:** Physical activity profile of Italian immigrants in Norway, by educational level (*n* = 531).

**Physical activity variables**	**Educational level**			**1-way ANOVA** ** (unifactorial model)**	**4-way ANOVA** **(multifactorial model)**
	**High-school** ** or lower** **(*n* = 205)**	**Bachelor's** **or Master's Degree** **(*n* = 234)**	**Doctoral** **Degree** **(*n* = 92)**		
**Perceived impact**	b, c	a, c	a, b	F_(2, 529)_ = 15.17 ^***^	F_(2, 515)_ = 20.40 ^***^
Negative	35.9%	10.2%	20.5%		
No impact	41.1%	39.5%	25.3%		
Positive	23.0%	50.2%	54.2%		
**Time spent sitting**	b, c	a, c	a, b	F_(2, 529)_ = 37.03 ^***^	F_(2, 515)_ = 71.79 ^***^
<6 h/day	60.5%	31.6%	16.3%		
6–9 h/day	36.1%	59.8%	71.7%		
≥ 10 h/day	3.4%	8.6%	12.0%		
**MVPA amount**	b, c	a	a	F_(2, 529)_ = 5.66 ^**^	F_(2, 515)_ = 9.29 ^***^
Never during the week	31.7%	18.4%	16.3%		
<150 min/week	25.4%	21.8%	21.7%		
150–300 min/week	28.3%	36.8%	35.9%		
> 300 min/week	14.6%	23.0%	26.1%		
**MVPA frequency**	b, c	a, c	a, b	F_(2, 529)_ = 5.73 ^***^	F_(2, 515)_ = 21.08 ^***^
M ± SD	2.30 ± 2.03	3.35 ± 2.16	3.51 ± 2.42		
**Green exercise**	b.c	a.c	a.b	F_(2, 529)_ = 6.94 ^***^	F_(2, 515)_ = 10.55 ^***^
Not on a weekly base	48.8%	29.5%	22.8%		
On a weekly base^d^	51.2%	70.5%	77.2%		
**Active transport**	b.c	a	a	F_(2, 529)_ = 7.56 ^***^	F_(2, 515)_ = 14.75 ^***^
Not on a weekly base	52.7%	29.5%	25.0%		
On a weekly base^d^	47.3%	70.5%	75.0%		
**Exercise in the gym**	b.c	a	a	F_(2, 529)_ = 6.77 ^***^	F_(2, 515)_ = 19.02 ^***^
Not on a weekly base	70.7%	49.6%	46.7%		
On a weekly base^d^	29.3%	50.4%	53.3%		
**Occupational MVPA**	b.c	a	a	F_(2, 529)_ = 9.19 ^***^	F_(2, 515)_ = 20.24 ^***^
Not on a weekly base	65.4%	76.9%	83.7%		
On a weekly base^d^	34.6%	23.1%	16.3%		
**Organized sports**	b	a	n.s.	F_(2, 529)_= 2.52 ^*^	F_(2, 515)_ = 3.81 ^*^
Not on a weekly base	91.2%	81.2%	85.9%		
On a weekly base^d^	8.8%	18.8%	14.1%		
**Other**	n.s.	n.s.	n.s.	F_(2.529)_ = 1.98	F_(2, 515)_ = 4.98
Not on a weekly base	69.3%	59.4%	54.3%		
On a weekly base^d^	30.7%	40.6%	45.7%		

*1-way ANOVA = Educational level included as single factor; 4-way ANOVA = Educational level included as factor while correcting for other sociodemographic variables (Gender, Age, and Region of residence) in a full factorial 4-ways ANOVA.*

*** *p <0.001;*

** *p <0.01;*

* *p <0.05.*

a−c *χ^2^ test for independence showed a significant difference (p <0.05): (a) vs. group 1; (b) vs. group 2; (c) vs. group 3.*

d *Original levels “Less than half of total MVPA,” “About half of total MVPA,” “More than half of total MVPA,” and “I only do this activity” are presented conflated*.

The multifactorial ANOVA identified also a number of significant interactions among the sociodemographic characteristics for several PA variables, herewith presented. A significant Gender by Educational level interaction was found for MVPA amount [F_(1, 515)_ = 6.35; *p* = 0.01] and Exercise in the gym [F_(1, 515)_ = 5.32; *p* = 0.02]. The Age by Region of residence interaction was significant for Perceived impact of moving [F_(1, 515)_ = 6.87; *p* = 0.01], Time spent sitting [F_(1, 515)_ = 4.04; *p* = 0.05], and Active transport [F_(1, 515)_ = 10.56; *p* <0.001]. A significant Age by Educational level interaction was found for the MVPA mode “Other” [F_(1, 515)_ = 5.55; *p* = 0.02]. Region of residence by Educational level interaction was significant for Perceived impact of moving [F_(1, 515)_ = 6.61; *p* = 0.01], Green exercise [F_(1, 515)_ = 8.19; *p* <0.001], and Occupational MVPA [F_(1, 515)_ = 12.27; *p* <0.001]. A significant interaction among Gender, Age, and Region was found for Exercise in the gym [F_(1, 515)_ = 4.51; *p* = 0.03] and Occupational MVPA [F_(1, 515)_ = 7.39; *p* = 0.01]. The interaction among Gender, Age, and Educational level was significant for MVPA amount [F_(1, 515)_ = 4.40; *p* = 0.04]. Finally, a significant interaction among Gender, Regin of residence, and Educational level was found for Organized sports [F_(1, 515)_ = 5.83; *p* = 0.02].

## Discussions

### Summary of the Main Findings

The purpose of this study was to investigate the extent to which Italian immigrants living in Norway perceive that moving to Norway had a negative or positive impact on their PA habits, as well as examine the PA profile of the Italians in comparison with the general Norwegian population. Furthermore, the study aimed to investigate whether and how the PA profile of the Italians was associated with key sociodemographic characteristics. The following main findings emerged:

RQ1. A large majority of Italian immigrants perceived they were as or more physically active in Norway than they would have been if they continued living in Italy, though as many as 20% of the Italian immigrants perceived a negative impact on their PA habits.
RQ2. No significant differences were found for any of the variables of PA levels (Sitting time, MVPA amount, and MVPA frequency) between the Italians and the Norwegian population, though some differences were found in relation to specific MVPA modes (e.g., active transport and exercising in the gym were more prevalent among the Italian immigrants than the Norwegian population).
RQ3. Compared with women and those with higher educational levels, men and those with lower educational levels tended to perceive a more negative impact of moving, be less physically active, and engage less frequently in all MVPA modes. Although generally more active, the Italians with higher educational levels were at the same time more sedentary (i.e., they spent more time sitting during a regular day and engaged in less occupational MVPA) than those with lower educational level. Compared with those living in the most urbanized region of Norway (Oslo and Akershus), a larger proportion of those who lived in less urbanized regions perceived a negative impact of moving. However, after controlling for multiple factors, no significant difference were found between the two groups with respect to their PA profile. Some, less consistent, differences were found also among age groups: while the prevalence of perceiving a positive impact decreased with increasing age, those aged 30–39 years reported the higher MVPA frequency as compared to all other age groups, with no differences for sitting time and MVPA amount being detected. Moreover, while exercising in the gym was less prevalent in the oldest age group (≥50 years), “other” activities were increasingly prevalent with increasing age.

In the following paragraphs, these findings are discussed in light of acculturation, gender dynamics, and social gradient.

### Increased Physical Activity as a Part of the Acculturation Into Norwegian Lifestyle

It has been previously suggested that the physical and cultural environment in Norway is, compared to other countries, particularly supportive for PA. Indeed, in Norway, there is generally good accessibility to safe and PA-supportive natural environments (e.g., well-lighted parks and walking/skiing trails in neighbor forests), together with a lively cultural atmosphere that values PA, sports, and outdoor recreations (24). Moreover, compared with other countries, urban settlements in Norway are generally small, with relatively little car traffic, and rather good levels of walkability and perceived safety (25). In spite of this supportive environment, evidence exists indicating that some immigrant populations, and women in particular (26–28), encounter barriers for engaging in PA. For this reason, it is remarkable to observe the relatively high PA levels of the Italian immigrants, as well as the large proportion of Italian immigrants perceiving that they become more active after moving to Norway. Interestingly, the Italian immigrants in Norway appear to be more physically active than their compatriots living in Italy. For instance, a 2015 report on the PA habits of the Italian general population indicates that 39% of Italians never engage in any sports or PA in their leisure time. This is a considerably larger proportion compared to the 23% of Italian immigrants in this study reporting not to engage any PA during a regular week (29). Moreover, only 24% of the Italian general population engage in some sports on a regular base, with 27% engaging in some form of unstructured PA such as walking or biking. This is, altogether, a considerably smaller proportion compared to the 77% of the Italian immigrants engaging in some MVPA during a regular week. It should be noted, however, that it is challenging to make direct comparisons between the findings of this study with the figures for the Italian general population, as these employed different measurements of PA.

The relatively high PA levels of Italian immigrants in Norway, as compared with the Italian and the Norwegian general populations, can be interpreted under the prism of acculturation. Acculturation can be defined as “the process of cultural and psychological change that takes place as a result of contact between cultural groups and their individual members” (30). In this perspective, an interesting case may be especially provided by the high prevalence of weekly green exercise participation, which was fairly equivalent among Italian immigrants as among the Norwegian general population. Green exercise and outdoor recreations are part of a lively cultural heritage in Norway, also known as *friluftsliv* (31). Activities such as walking in the forest or on the mountains (or cross-country-ski trips, during the winter) are common activities during the weekend or holiday periods. Figures from Statistics Norway show for instance that, in 2020, 79% of adult Norwegians have reported doing short walks in the forest or on the mountains, with 52% having done longer hiking tours (32). Per counter, green exercise is much less participated in Italy than in Norway: in 2015, only 25% of Italians reported doing PA outdoors in urban settings (parks but also city streets, etc.), while 31% visited natural environments outside the city (mountains, sea- or lake-shores, forests, etc.) (29). Unfortunately, to the best of the authors' knowledge, figures on *weekly* green exercise participation in Italy are not available. However, figures from other European countries exist, indicating that outside Norway (or other Scandinavian countries) green exercise participation is not as common. For instance, a 2016 study from England found that only 20% of the population perform “active visits” in natural environments of a duration of at least 30-min during a regular week (33).

It should be noted that the evidence presented above, which suggests that the Italian immigrants underwent an acculturation process relative to their PA habits, is likely associated with the generally high SES of this group. Indeed, in line with figures from the Italian Embassy in Norway (22), 61% of the sample in this study had a University degree (i.e., a Bachelor's, Master's, or Doctoral degree) as opposed to 35% of the general Norwegian population (34) and 15% of the Italian general population (35). As an individual educational level is a known determinant of PA participation, this may contribute explaining the greater adaptability of the Italians immigrants to the new socio-cultural context, as well as their relatively high PA levels. Higher skilled people have been previously shown to be more likely to dive into a new culture and cope better the transition into a new environment (36). Structural aspects may also be taken into consideration. For instance, people with a lower educational level may be employed in jobs that require less specialized skills (e.g., shops or restaurants), which typically have tiring working schedules offering less opportunities for engaging in leisure-time PA. The higher prevalence (relative to the Norwegian population) of active transport and gym-based exercise among the Italian immigrants is also in accordance with such perspective, as these activities tend to be more common among Norwegians with higher SES (14). On the other hand, it should be noted that exercising in the gym (such as group-exercise sessions, fitness and weight lifting) is the most popular mode of PA in Italy (29), so the different patterns between Norwegians and Italian immigrants with respect to this specific activity may be also explained by cultural preferences.

### A Gender Perspective on the Physical Activity Profile of Italian Immigrants in Norway

In general, in Italy (as well as many other countries), women tend to be less physically active than men (29). Figures for the Italian general population show, in fact, that even though slightly more women than men (29 and 24%, respectively) engage in unstructured forms of PA such as walking or biking for transport purposes, a much larger prevalence of men than women (30 and 20%, respectively) practice sports or structured exercise on a regular base (29). This is in contrast with figures from Norway, which indicate near-to-equal PA levels for men and women (see section 1.2). If on the one hand this greater PA and sport participation among women in Norway can be associated with a more supportive environment, and especially a generally greater perceived safety (see section 4.2), this is also a phenomenon deeply embedded in the Norwegian socio-cultural context (24, 37).

In this study, the PA levels of the Italian immigrant women appear to be more in line with the patterns reported for the Norwegian population than those reported for the Italian general population. Indeed the findings are rather indicative of a gender-gap *in favor* of the Italian immigrant women, a pattern that might be partly explained by the fact that they were (in average) younger and more highly educated than their male counterparts (see the medium-sized correlations of gender with age and educational level in Supplementary Figure 1). Alongside the finding that a large proportion of the Italian immigrant women perceived being more active in Norway than they would have been if they continued living in Italy, this suggests that many Italian immigrant women changed their PA habits and aligned with the patterns of the Norwegian population. While this finding indicates an acculturation to values and practices specifically related to PA (as discussed above), on the other hand, it may also be indicative of an acculturation into gender roles that are more common in the Norwegian context. In Norway, gender gaps are less remarkable than in Italy (38, 39). Recent figures published by Statistic Norway indicate positive trends toward a more equitable balance between genders in relation to family tasks (40, 41). Moreover, as average weekly working hours are shorter in Norway than in Italy (42), the greater availability of time to practice PA coupled with cultural norms sustaining women participation in sports is likely to contribute explaining why Italian immigrant women experienced a positive change of their PA habits in Norway.

### The Importance of Understanding Social Gradients Within Immigrant Populations

The prevalence of Italian immigrants perceiving a negative impact on their PA habits after moving to Norway was relatively small, but not-negligible (20%), especially considering that this prevalence was even larger in sub-groups that are typically more vulnerable when it comes to complying with health-related behaviors (i.e., older individuals and those with lower SES). Similarly, the Italians' PA levels varied in relation to different sociodemographic characteristics to an extent that is broadly in line with known patterns of PA behaviors (8–10). Thus, although the Italian immigrants emerge as a relatively active population, efforts are still needed in order to promote greater PA participation targeting the most vulnerable groups. In particular, in line with a health promotion perspective, more attention should be given on understanding who and why perceive a negative impact on their PA habits, as well as who and why have insufficient PA levels, so that effective initiatives can be designed and implemented to promote PA among those who need it most.

In the present study, the relationship of PA with age appears somewhat complex, and not fully in line with the existing evidence. It should be noted, however, that also the literature on age-related gradients of PA in the Norwegian population is somewhat inconsistent. For instance, findings from the 2019 Survey on living conditions indicate that the prevalence of individuals engaging in MVPA for at least 150 min during a regular week decreases progressively across age groups, ranging from 67% among those aged 16–24 years to 46% of those aged 67 years or older (12). This is not consistent with the findings of a 2015 report by the Norwegian Ministry of Health (13), which found no significant differences across age groups in the extent to which Norwegians meet the PA recommendations as based on self-reported assessments. Findings from the same report based on objective assessments of PA (i.e., accelerometry) indicate instead that the prevalence of individuals meeting the PA recommendations was greater among those aged 50–64 years as compared with all other age groups. When it comes to understand the relationship of PA with age, however, it is probably important to consider the specific modes of MVPA in which individuals engage. Indeed (in line with previous studies on both the Italian [28] and the Norwegian population [14]), the findings of the present study indicate that individuals' preferences for specific activities vary across different age groups. This information is precious, as previous studies have emphasized the importance of proposing PA initiatives tailored on specific groups of immigrants based on their preferences and cultural affinity (43). In the present study, the prevalence of perceiving a negative impact increased with increasing age, thought a matching pattern was not observed in relation to PA levels, which may suggest a deterioration of the PA habits with respect to subjective standards. Alongside the fact that PA is, in general, a fundamental element of active aging, this indicate the importance of designing PA initiatives specifically tailored for older Italian immigrants. Among the older Italian immigrants, “other” modes of MVPA were especially prevalent. Unfortunately, no additional information is available to understand more in depth what this category of activities exactly entails, so more in-depth investigations are recommended.

The finding relative to region of residence are in line with patterns observed in the Norwegian population, thought they deviate from Italian figures. In fact, while in Italy PA levels tend to be higher in more urbanized and densely populated areas [for instance, in the Northern regions and in larger urban settings (29)], in Norway there appears not to be significant difference in relation regions or population density (13). This could be (at least in part) explained by the fact that, in Norway, PA-supportive environments (parks, trails in forests and maintains, coasts, etc.) are generally abundant and accessible both in more as well as less central areas. While the greater prevalence of occupational MVPA observed among the Italian immigrants is in line with figures for the Norwegian population (14), among the Italians no differences were observed for other modes of PA that are typically more common in more central areas, such as active transport and exercising in the gym (14). This could be explained by the fact that, in general, these activities were more prevalent among the Italian immigrants than the Norwegian population. On the other hand, it should be noted that the variable “region of residence” used in this study might have not been able to detect more nuanced differences relative to centrality, since it did not fully discriminate among individuals living in urban (large or small) v. rural settings.

The relation of PA with educational level (the most consistent and pronounced, as compared with the other sociodemographic variables in this study) was in line with patterns previously observed in both, the Norwegian (13) and the Italian (29) general populations (see section 4.1). Interestingly, while PA levels were positively associated with educational level, so was sitting time, indicating that the Italians with a higher educational level were more active, but also more sedentary. This may be explained by the fact that more highly educated individual are more likely to be employed in sedentary (e.g., office-based) jobs. While this is not always the case among immigrant populations, it is facilitated within the context of EEA migration, and thus among Italian immigrants in Norway, through the Bologna Process [which, among other things, facilitates mutual recognition of study qualifications earned abroad within the EEA (44)]. In the past decades, studies have emphasized how PA and sedentarism are distinct health-related behaviors, each independently predictive of health outcomes (1, 2). Thus, this finding indicates the importance of designing different initiatives for immigrants with different SES.

### Strengths and Limitations

To the best of the authors' knowledge, this is one of the few studies focusing on intra-EEA immigration groups, and the first study specifically investigating PA patterns among Italian immigrants. The findings of this study provide new knowledge about an under-researched immigration group, but also shed light on factors influencing health behaviors among immigrant populations in general. By using instruments developed in line with Norwegian recurrent surveys, we could make direct comparisons between the PA habits of Italian immigrants and the Norwegian population. Another strength of this study is the inclusion of a measurement of sedentary behavior (i.e., Time spent sitting). A recent systematic mapping review found, indeed, that only few studies have investigated factors associated with this variable among immigrant populations within the European context (45). Furthermore, by applying a resampling approach, we were able to balance the dataset in line with key sociodemographic characteristics of the Italian immigrants in Norway, as provided by official sources. The study presents, however, a number of limitations that ought to be taken into account.

#### Representability of the Sample and Generalizability of Findings

The resampling process permits to balance the dataset of a subset of responders in accordance with key variables observed in the original population (i.e., the Italian immigrants in Norway, as reported by official sources). ADASYN allows doing this by creating artificial cases reflecting the pattern of the authentic cases detected in the dataset. The data created are, thus, expected to accurately simulate the actual respondents' answers. The main limitations of such oversampling approach is that it could overestimate patterns observed in small subgroups, inducing a low ability of this class generalization. Fortunately, the dataset used in this study does not appear to have suffered from this problem.

#### Instruments' Validity

Another limitation of the study related to the instrument used to assess the Italians general PA levels. In particular, the measure of MVPA amount conflates light-to-moderate intensity and vigorous intensity PA. This may lead to an under-estimation of the prevalence of individuals meeting the recommended PA levels by excluding those who achieve sufficient PA levels through vigorous-intensity PA (which, in line with the WHO guidelines, has a cut-off of at least 75 min/week). The MVPA variable used in the present study is, however, a commonly used indicator of PA levels in population studies, including the Norwegian Survey on living conditions, which served as a comparison in this study. It should be also noticed that, differently than previous recommendations (46), the most recent WHO recommendations removed the reference to the 10-min bouts (7), which was instead present in the instrument for Mens Sana in Corpore Sano study. The figures used in this study to compare the Italians' MVPA modes with the Norwegians' were retrieved from the study by Calogiuri et al. (14), which was in turn based on a national survey conducted in October 2012: i.e., six and a half years earlier than the data collection for this study. This different time, as well as the different season, in which the data for MVPA mode were collected may have led to some inaccuracies in the comparison between the Italians and the Norwegians.

#### Analytical Approach

As the purpose of this study was largely descriptive, with a focus on comparing the PA patterns of Italian immigrants with the Norwegian general population, the analytical approach was rather simple. This is likely to have limited the understanding of the phenomenon in its complexity. Moreover, only a selected number of sociodemographic variables were presented. This was a deliberate choice, partly motivated by the relatively small sample size, as well as the intention to provide outcomes easily comparable with existing evidence [e.g., national reports on PA patterns often present only bivariate analyses, see e.g. (13, 29)]. It should be also noted that the choice of PA correlates for this study emerged both from a solid theoretical underpinning (i.e., selection of known determinates of PA behaviors) as well as a preliminary exploration of the data.

A possible important limitation, however, is the fact that the present study did not include the duration of residence (i.e., the number of years passed since the Italians migrated to Norway) as a variable in the analyses. Duration of residence may be indicative of acculturation, and it has been previously found to be a factor that may influence PA behavior in immigrant populations in Europe (45). However, the way in which duration of residence influences the immigrants' PA behavior is little clear. For instance, while some studies did not find a relevant impact of this variable on immigrants' PA [see e.g., (47)], other studies found significant, though not consistent, associations. For instance, Dawson et al. (48) found a positive association between time since migration and PA among immigrant women (but not men) in Sweden, with the immigrant women's PA levels increasing with increasing duration of residence. On the other hand, a study on Tunisian immigrant men in France found lower PA levels among immigrants who resided in the country for ≥ 30 years as compared with those who resided in the country for shorter time, thought the effect was no longer significant when adjusting for age at migration (49). In the present study, the choice of not including duration of residence was mainly motivated by the fact that preliminary analyses found it to be highly correlated with the Italian immigrants' age (see Supplementary Figure 1). While this is indicative of the fact that most respondents had similar age at time of migration, it makes it difficult to discern any effect of duration of residence from possible effects of age itself. It was thus decided to base the analyses only on age, so that the patterns of the Italian immigrants could be compared with those of the Norwegian population and the Italian nationals. This implies, however, that the patterns presented in relation to age, may be influenced by the duration of residence and, more in general, acculturation.

## Conclusions

The findings of the present study indicate that a large majority of Italian immigrants in Norway perceived they were as or even more physically active in Norway than they would have been if they continued living in Italy. However, the prevalence of perceiving a negative impact was greater in specific sub-groups (the men, older individuals, those who live in less urbanized regions of Norway, and those with lower SES). No significant differences between the Italian immigrants and the general Norwegian population were found for key indicators of PA levels, though some differences were observed in relation to specific activities. Associations of PA with different sociodemographic characteristics were observed, especially in relation to gender, educational level and, to a certain extent, age. In contrast with patterns observed in the Italian general population (as well as patterns observed in other immigration groups), women were generally found to be more physically active than men. These findings shed light on the PA habits of Italian immigrants living in Norway, a relatively small but rapidly growing immigration group and can be used to inform initiatives to promote PA in this or similar immigrations groups. In spite of some limitations, this study indicates the potential of expanding the research on health and PA to under-researched immigrants groups, in particular within the EEA context. As mobility within the EEA is on the rise, it is important to understand how individuals interact with the opportunities and the culture of the country of resettlement, as well as how social gradients influence PA patters in the context of migration.

## Data Availability Statement

The datasets generated for this study is not publicly available at the moment. All relevant aggregated data are provided in the paper. The data used in the current study may be made available after agreement with the authors.

## Ethics Statement

Ethical review and approval was not required for the study on human participants in accordance with the local legislation and institutional requirements. The patients/participants provided their written informed consent to participate in this study.

## Author Contributions

GC drafted the overall manuscript and led the team of authors. AR performed the resampling and statistical analyses and drafter the relative parts in the Methods. LT was the overall project coordinator for the Mens Sana in Corpore Sano project and contributed substantially to the design of the study as well as the revision of the intellectual content. All authors contributed to the article and approved the submitted version.

## Conflict of Interest

The authors declare that the research was conducted in the absence of any commercial or financial relationships that could be construed as a potential conflict of interest.
