# Supplementary material for: Physical Activity Levels and Perceived Changes in the Context of Intra-EEA Migration: A Study on Italian Immigrants in Norway
Source: Front Public Health. 2021 Jul 7;9:689156. doi: 10.3389/fpubh.2021.689156 (PMC8294772; doi:10.3389/fpubh.2021.689156)
Supplement: Supplementary file 1 [file Data_Sheet_1.pdf]

Supplementary Material

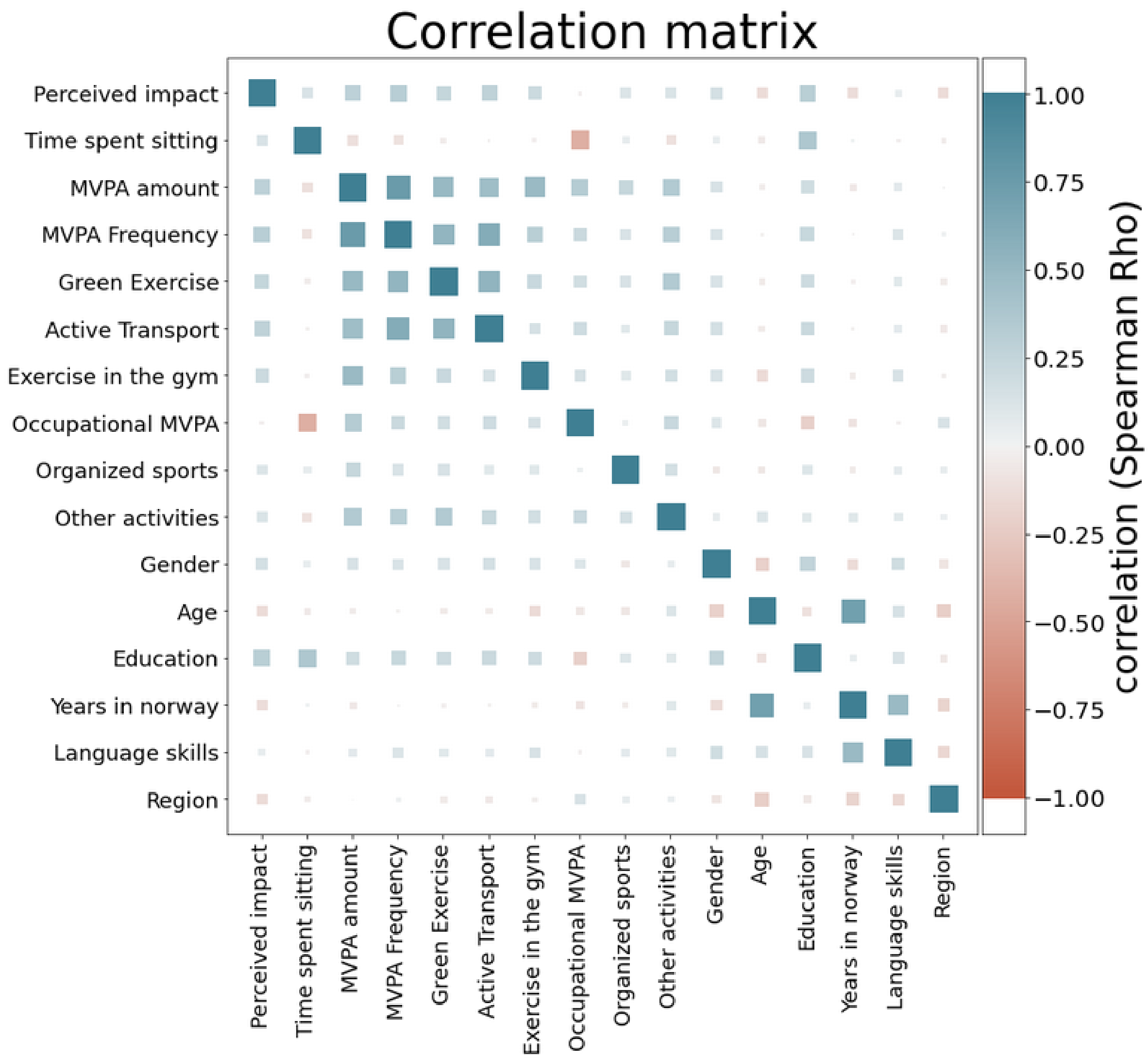

Supplementary Figure 1. Correlation matrix. The dimension of the squares refers to the strength of the relationship between two variables. Blue and red colours refer to the direction of the correlation (i.e., positive and negative, respectively) and, in accordance with the dimension, the darker is the color the strength is the correlation.

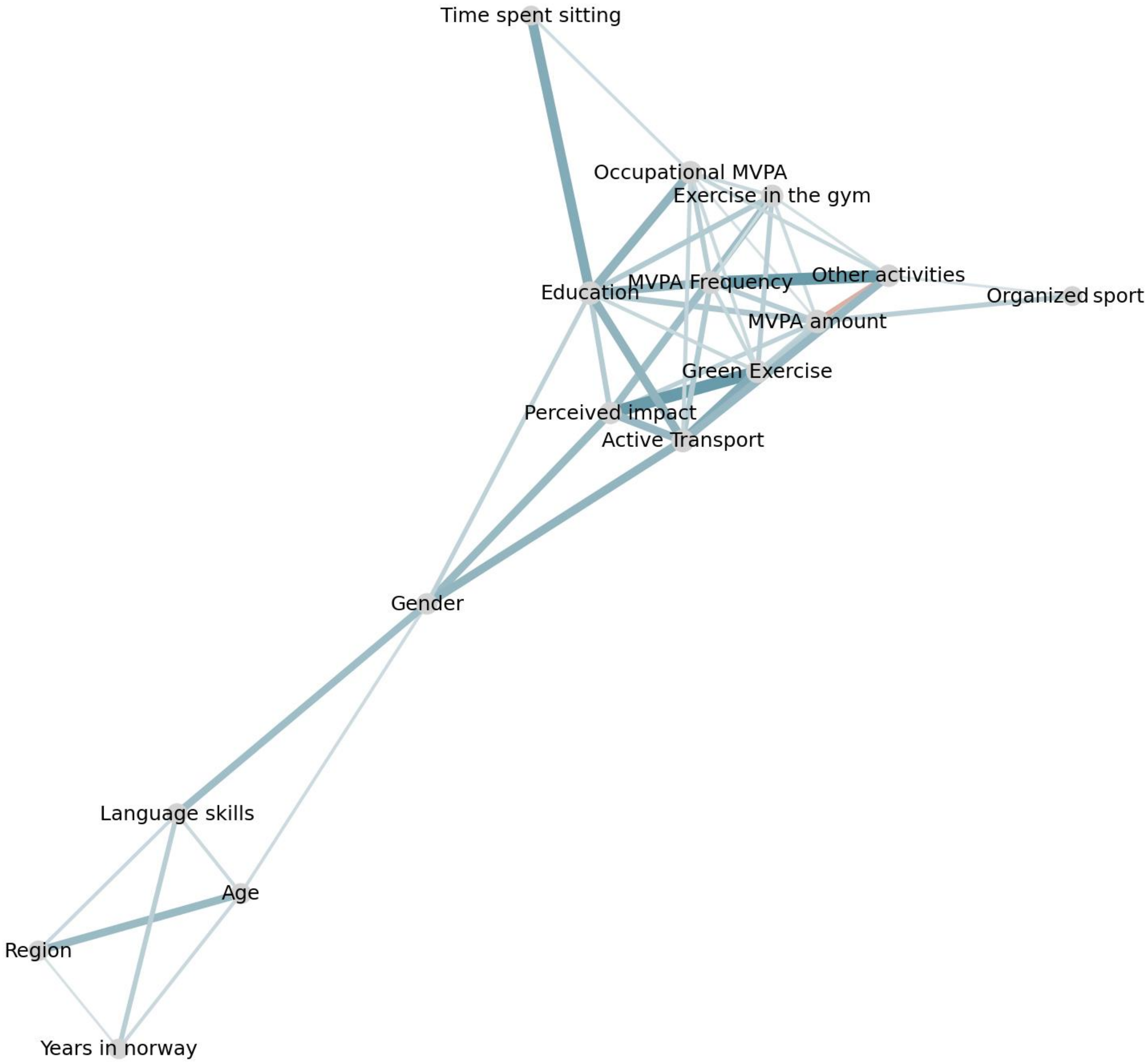

Supplementary Figure 2. Correlation network (number of nodes = 15; number of edges = 44; network density = 0.41). In this figure, each node refers to a specific variable, while the links are defined by the relationship between two nodes. The colour and the dimension of each link refers to the strength of the correlation. In particular, red and blue links refer to negative and positive relationships, respectively. The darker and the bigger is the links, the higher is the correlation between the variables. The position of each node is defined as the similarity among features based on correlation coefficients. Supplementary Table 1 shows the importance of each feature in the network expressed as node degrees (number of links), betweenness centrality (measure of centrality for each variable) and degree centrality (the fraction of nodes it is connected to). The colours of the links refers to the correlations between nodes as showed in the correlation matrix (Supplementary Figure 1).

Supplementary Table 1.

Correlation network metrics. In order to assess the centrality of each node, the table presents the node degrees (number of links), betweenness centrality (measure of centrality for each variable) and degree centrality (the fraction of nodes it is connected to) for each node.

Variables	Node degree	Between centrality	Degree centrality
MVPA amount	9	0.09	0.60
Education	9	0.19	0.60
MVPA Frequency	8	0.01	0.53
Green Exercise	8	0.01	0.53
Active Transport	8	0.15	0.53
Occupational MVPA	8	0.04	0.53
Perceived impact	7	0.08	0.47
Exercise in the gym	7	0.01	0.47
Other activities	7	0.04	0.47
Gender	5	0.42	0.33
Age	4	0.11	0.27
Language skills	4	0.11	0.27
Years in norway	3	0.00	0.20
Region	3	0.00	0.20
Time spent sitting	2	0.00	0.13
Organized sports	2	0.00	0.13
